# Anatomic variant of the inferior lateral cutaneous branch of the radial nerve during the posterior approach to the humerus: a case report

**DOI:** 10.1186/s13037-015-0063-8

**Published:** 2015-05-14

**Authors:** Li Sun, Brian K Park, Salil Gupta, John T Capo, Richard S Yoon, Frank A Liporace

**Affiliations:** Division of Orthopaedic Trauma, Department of Orthopaedic Surgery, Jersey City Medical Center, Jersey City, NJ USA; Division of Orthopaedic Trauma, Department of Orthopaedic Surgery, NYU Hospital for Joint Diseases, New York, NY USA; Division of Hand and Upper Extremity Surgery, Department of Orthopaedic Surgery, NYU Hospital for Joint Diseases, 301 E 17th Street, Suite 1402, New York, NY 10003 USA

**Keywords:** Anatomic variant, Radial nerve, Lateral cutaneous branch, Humerus fracture

## Abstract

Iatrogenic injury during the posterior approach to the humerus during operative fixation is not an uncommon occurrence. A comprehensive understanding of the normal anatomy and its variants is of paramount importance in order to avoid such injury. Typically, the inferior lateral cutaneous branch of the radial nerve originates towards the distal end of the humerus at the inferior portion of the spiral groove. Here, we report an important variant of this nerve, which originated significantly more proximal than expected, further emphasizing the importance of identification, dissection and protection of the radial nerve and its major branches.

## Background

Understanding normal anatomy and being aware of potential variants is of paramount importance during the operative fixation of fractures. Especially when regarding the radial nerve, injury can occur despite a comprehensive understanding and meticulous dissection [1]. While there have been numerous clinical and cadaveric studies investigating anatomical patterns of the radial nerve, none have described a high-branching variant of the inferior lateral cutaneous nerve [2-9]. Here, we present a case of this anatomic variant we encountered during the posterior approach of humerus during operative fixation.

## Case presentation

**Figure 1:**
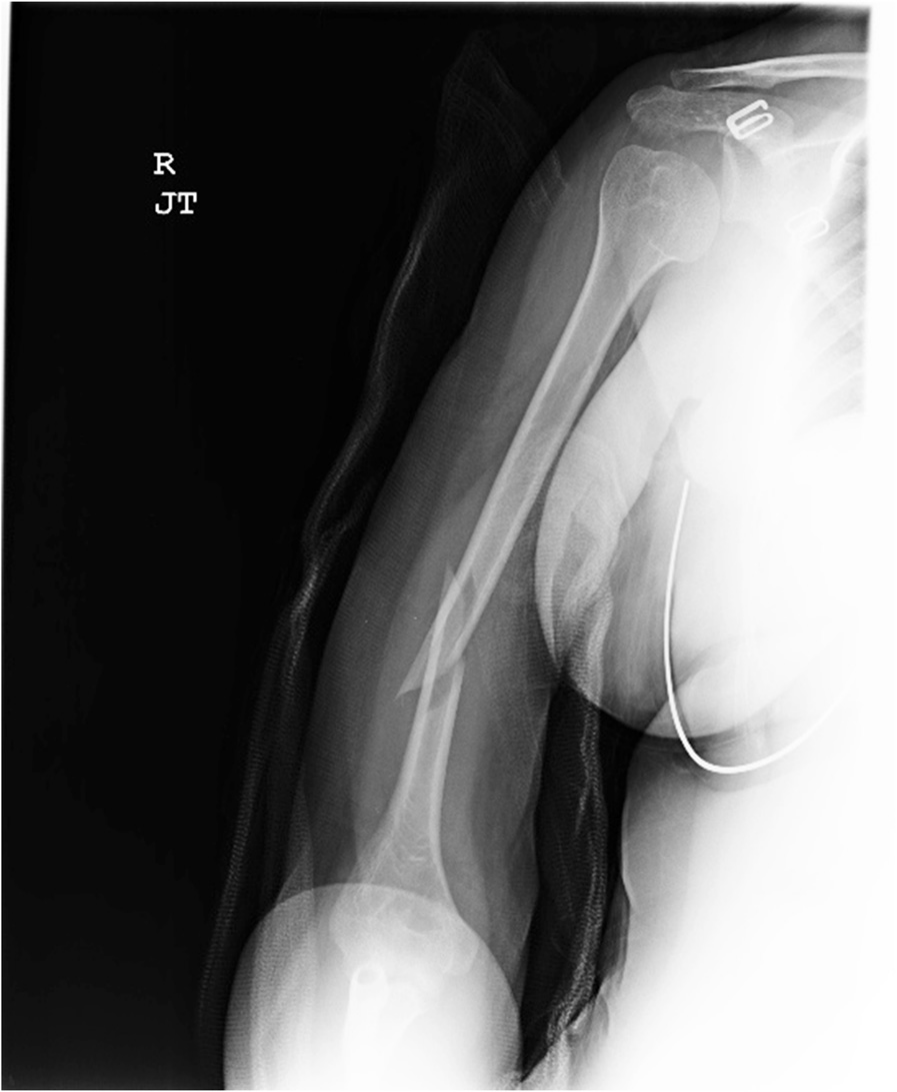
Anteroposterior (AP) view of the humerus at 6 weeks exhibiting malalignment and minimal callus formation.

Patient is a 27 year-old right hand dominant female who presented initially following an assault where a car door was closed on her right arm. She was evaluated and treated at a local emergency department with a coaptation splint. She had sustained a closed injury and remained neurovascularly intact. Initial management consisted of conversion over to a fracture brace and close follow-up. However, six weeks from injury, the patient endorsed continued pain. Examination revealed tenderness and movement through the fracture site; radiographs revealed unacceptable angulation and minimal callus formation (Figure 1). With persistent pain, movement through the fracture and minimal healing, the patient was indicated for operative fixation.

**Figure 2:**
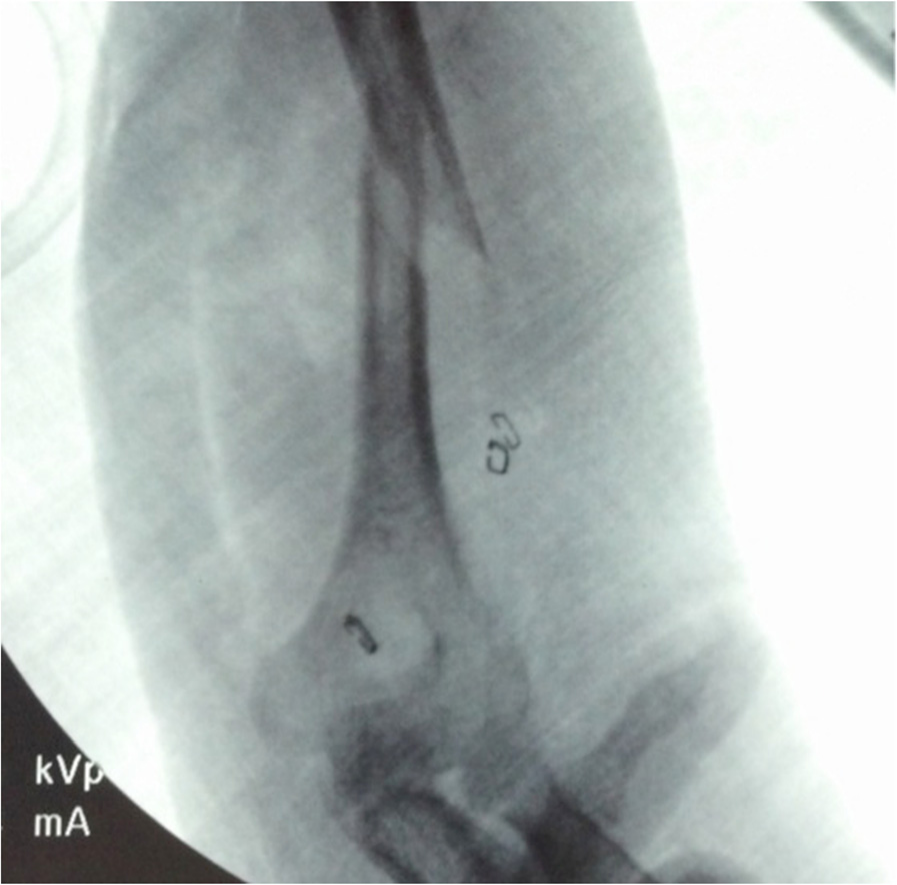
Stress fluoroscopic image noting gross motion at the fracture site.

**Figure 3:**
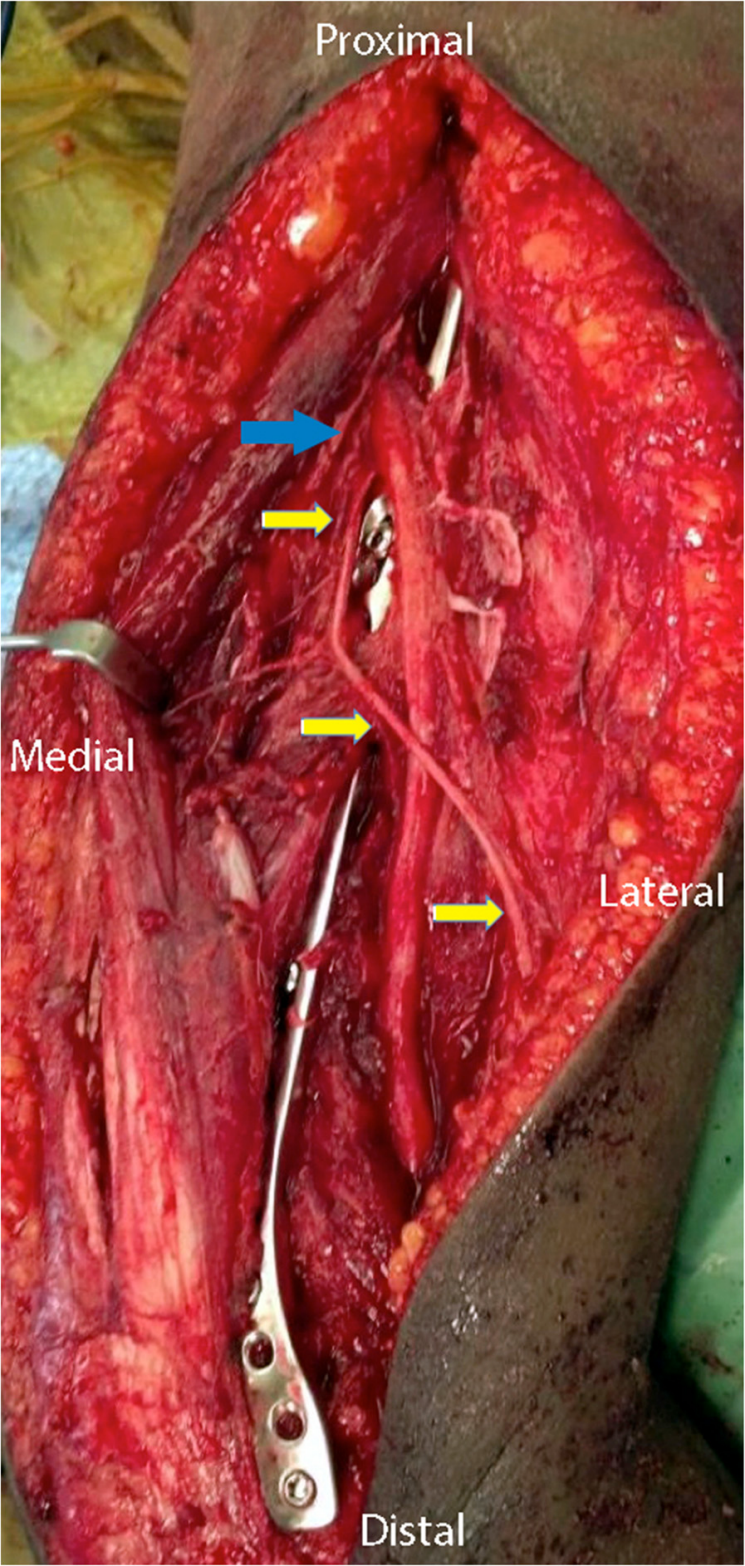
Via the posterior approach to the humerus, the inferior lateral cutaneous branch of the radial nerve was traced back to a very high origin (arrows), an anatomical variant from its typical branching location, which would typical be at the inferior/distal end of the spiral groove.

**Figure 4:**
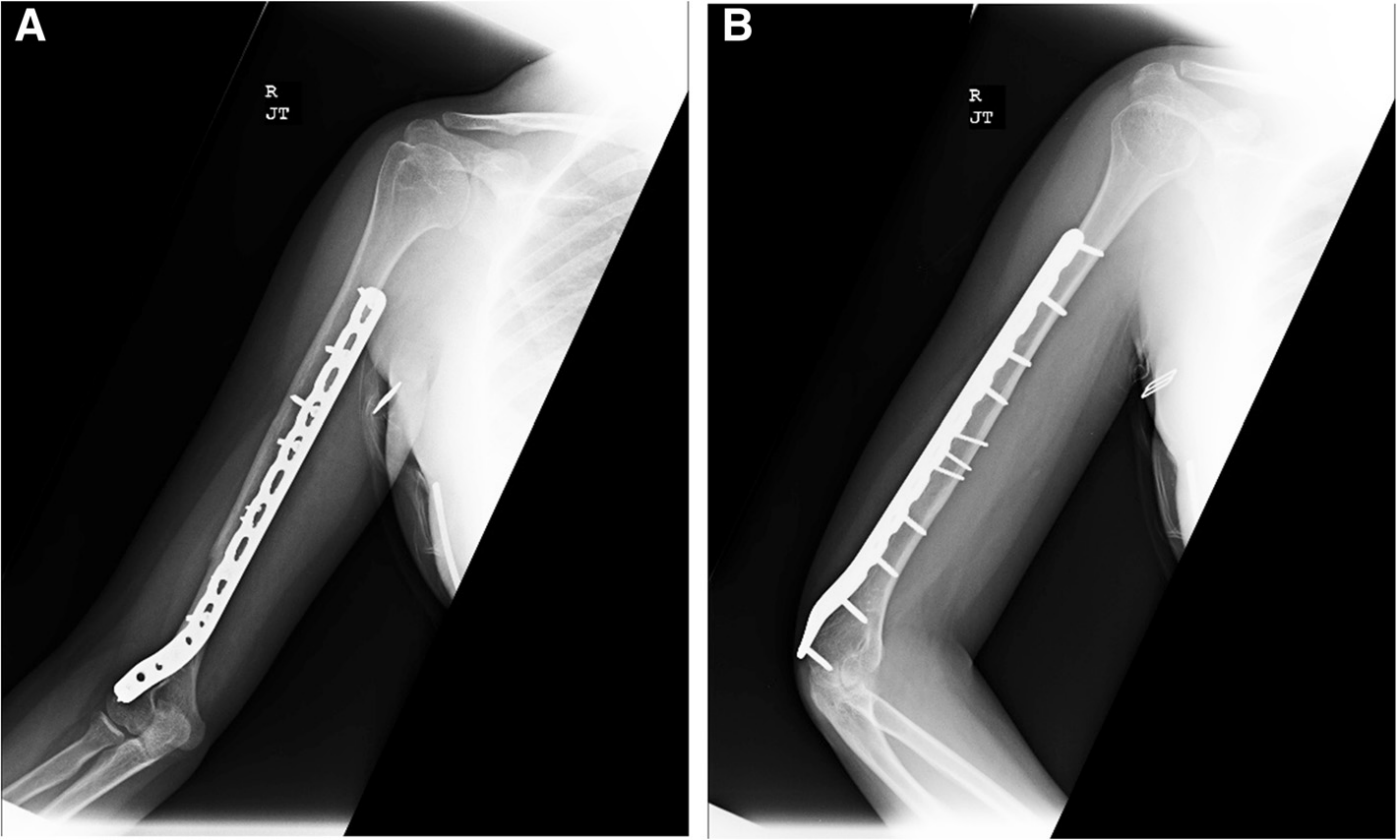
**A-B** AP and lateral radiographs of the healed right humerus fracture approximately 1 year following operative fixation.

The patient was brought to the operating room and placed in the lateral decubitus position. Stress fluoroscopy exhibited gross motion at the fracture site (Figure 2). Via the posterior approach to the humerus, the radial nerve was found approximately two fingerbreadths above the confluence of the triceps lateral head, long head and aponeurosis [8]. Once the main trunk of the radial nerve was found, the surrounding branches were carefully isolated and dissected. The inferior lateral cutaneous branch was found subcutaneously and traced back to its origin (Figure 3). This origin was noted to be a very proximally branching variant of the inferior lateral cutaneous branch, which is typically found at the inferior/distal end of the spiral groove, near the metadiaphyseal junction of the distal third of the humerus [10]. Operative fixation concluded without complication and the wound was closed primarily. The patient healed without complication and at 1-year follow-up was noted to have near full range of motion of the elbow (10-135) and was without complaints (Figure 4A-B).

Iatrogenic nerve injuries are among the devastating complications in the treatment of humerus fractures. Recent studies showed the rate of iatrogenic nerve injury in operatively treated supracondylar humerus fractures is 3% to 4% [11]. The iatrogenic radial nerve injuries during surgical treatment of humeral shaft fracture could be well over 4% [1]. With a relatively high rate of potential injury, careful identification and dissection of the nerve and its branches is of paramount importance. Studies of radial nerve with its anatomic location and relationship to the surrounding soft tissue and bony structure provide great guidance for the surgical approach in the treatment of humerus fracture. The radial nerve originates from the posterior cord of the brachial plexus passing through the spiral groove on the posterior aspect of the humerus. The radial nerve enters the upper arm through the spiral groove between the lateral head and the medial head of the triceps muscle. Branches in the spiral groove include, posterior cutaneous nerve of the arm, inferior lateral cutaneous nerve of the arm, posterior cutaneous nerve of the forearm, branch to lateral head of triceps, branch to medial head of triceps and anconeus. The 1st branch is the posterior cutaneous nerve of the arm at the groove level. The 2nd branch is the inferior lateral cutaneous nerve of the arm. The inferior lateral cutaneous nerve of the arm arises from the radial nerve at the lower part of the spiral groove, at a mean of 14.2 cm proximal to the lateral epicondyle [10]. After the radial nerve passes through the spiral groove of the humerus, it then enters the anterior compartment of the arm.

The inferior lateral cutaneous nerve of the arm is the branch of radial nerve that provides sensory and vasomotor innervations to the lower, lateral aspect of the arm. Understanding the variants of sensory nerve not only helps in the identification of radial nerve, but also reduces the chance of iatrogenic injury. In this case, the inferior lateral cutaneous nerve of the arm branched off at the level high above the spiral groove with a long arm branching down laterally into the subcutaneous tissue and skin. Hannouche et al, in a cadaveric study, noted the same takeoff origin of the inferior lateral cutaneous branch in all 18 specimens, which was at the inferior end of the spiral groove [10]. In our case, with such an abnormally proximal branching point of the nerve, this only reemphasizes the importance of careful dissection of the major nerve branches to avoid iatrogenic injury.

## Conclusion

Despite a seemingly reliable anatomic understanding the radial nerve and its branches, we report here an important variant of the inferior lateral cutaneous branch of the radial nerve. Typically, its origin is in the distal third of the humerus at the inferior end of the spiral groove, however, we report an abnormally high branching variant well above even the proximal extent of the spiral groove. We recommend using the confluence of the triceps lateral head, long head and aponeurosis [8] to identify radial nerve then trace proximally towards spiral groove to locate branches of radial nerve, including the possible anatomic variants. Identifying and reporting anatomic variants is essential to reemphasize the importance of dissection and protection of the major branches in order to avoid iatrogenic injury.

## Consent

Informed consent was obtained prior to the completion of this manuscript.
